# Confounding analysis with gaming aspects in predicting psychological distress of esports players

**DOI:** 10.1002/pchj.728

**Published:** 2024-02-01

**Authors:** Soon Li Lee, Cai Lian Tam

**Affiliations:** ^1^ Department of Psychology, Jeffrey Cheah School of Medicine and Health Sciences Monash University Malaysia Subang Jaya Malaysia

**Keywords:** confounding analysis, esports, internet gaming disorder, psychological distress

## Abstract

This research aimed to explore the interplay between the central and peripheral aspects of gaming and their correlation with internet gaming disorder (IGD) in predicting indicators of psychological distress. The study proposed that the core and peripheral characteristics of gaming serve as confounding variables affecting the direct relationship between IGD and psychological distress. Employing a longitudinal design spanning two distinct timeframes, the research involved 100 esports players from Malaysia, all of whom had participated in at least one official tournament. The outcomes revealed that both the core and peripheral attributes of gaming significantly predicted stress, anxiety, and depression. However, IGD exhibited a significant predictive association only with stress. Notably, the study also detected the confounding effects of core and peripheral characteristics in the direct link between IGD and stress. These results imply that core and peripheral gaming traits should be interpreted as individual differences that amplify susceptibility to IGD and its associated psychological distress indicators. Moreover, the findings suggested that IGD might not be a prominent concern for esports players, potentially due to their training in performance management as athletes. The paper explores further implications stemming from these findings.

## INTRODUCTION

In the recent fifth revision of the *Diagnostic and Statistical Manual of Mental Disorders* (DSM‐5; American Psychiatric Association [APA], 2013), internet gaming disorder (IGD) was introduced as a provisional diagnosis pending further research (Petry & O'Brien, 2013; Pontes & Griffiths, 2014). IGD is characterized by the persistent use of the internet for online gaming, causing significant distress or impairment over a nine‐month period (APA, 2013). Recognizing the need for robust research, various screening tools have been developed, such as those proposed by Pontes and Griffiths (2015). The World Health Organization (WHO, 2018a) has also acknowledged the consistent research findings regarding IGD and identified it as a potential clinical concern. Consequently, “gaming disorder” was included as a provisional term in the 11th Revision of the International Classification of Diseases (ICD‐11; WHO, 2018b). Empirically, gaming disorder has shown a higher prevalence in Southeast Asia (Chia et al., 2020; Kim et al., 2022), which has drawn significant public attention and concern in the affected regions (Chandy, 2019; The Star, 2021).

The introduction of IGD has prompted subsequent empirical investigations aiming to prove its concerning effects on various samples, including schoolchildren, deemed vulnerable by various stakeholders (Chen et al., 2021; Chen, Chen, Hou, et al., 2022), school teachers (Chen, Chen, Gamble, et al., 2022), esports players with high gaming intensity and proficiency (e.g., Bányai et al., 2020), and individuals misusing substances (Chen, Chang, Chang, et al., 2022). To shed light on this aspect, researchers have moved beyond cross‐sectional design, employing a longitudinal approach to illustrate the conceptual relationship between problematic gaming and various psychological outcomes (e.g., Chang et al., 2022). These empirical findings have revealed that the fixation on gaming activities is induced by certain internal predispositions, such as the existing fear of the infectious disease COVID‐19 (Chen et al., 2021). However, given the longitudinal nature of the design, the findings have illustrated the complex interplay between problematic gaming and psychological outcomes. While it is expected for individual differences to induce susceptibility to problematic gaming, as shown in previous studies (e.g., Chen et al., 2021), one longitudinal finding indicated the possibility for problematic gaming to consolidate the saliency of an internal predisposition, such as self‐stigma (Chen, Chang, Chang, et al., 2022). Further examination revealed that self‐stigma did not significantly predict problematic gaming (Chen, Chang, Chang, et al., 2022), contradicting established findings on internal predispositions as factors that induce problematic gaming. In general, problematic gaming induces susceptibility to psychological distress (e.g., Chang et al., 2022; Chen, Chen, Gamble, et al., 2022). However, these relationships may be conditional upon the situational context. As indicated previously, individuals with high levels of fear of COVID‐19 are more likely to sustain unregulated gaming behavior (Chen et al., 2021). Hence, it is sensible to expect the enhanced effect of problematic gaming on psychological distress during the period of the pandemic. However, during the peak period of COVID‐19, a decline in gaming time was observed among schoolchildren (Chen et al., 2021), possibly due to the shift to online learning, which dictated the time spent using technology (Chen, Chen, Hou, et al., 2022). This shift may have reduced the saliency of the effect of problematic gaming on psychological distress. Altogether, these longitudinal studies imply the need to consider the complex interplay between problematic gaming and psychological variables. It is also imperative to consider the characteristics of the sample involved, such as schoolchildren who have been distracted from prolonged gaming (e.g., Chen, Chen, Hou, et al., 2022). Hence, this research focuses on esports players who are highly motivated in gaming (Bányai et al., 2019a, 2019b, 2020; Martončik, 2015), which may be a cause of their susceptibility to psychiatric concerns (Bányai et al., 2019a, 2019b, 2020).

The surge in the popularity of esports, also known as competitive video gaming (Hamari & Sjöblom, 2017), has raised considerable concern among dedicated players, especially those engaged in competitive gaming. As of 2022, it was estimated that the total esports audience reached 532 million, and this figure is expected to steadily increase annually, potentially surpassing 640 million in the upcoming years (Newzoo, 2022). This burgeoning industry has been recognized for its lucrative revenue streams (e.g., Newzoo, 2022) and substantial earnings (e.g., Esports Earning, 2022), making it an enticing career prospect for tech‐savvy young adults (Bányai et al., 2019a, 2019b). Given their competitive nature, professional esports players adhere to strict training requirements similar to traditional athletes (Taylor, 2012), necessitating more time spent on gaming than casual players (Bányai et al., 2019a, 2019b). However, there is a notable conflict, as the profession's potential for attractive income poses risks to the well‐being of players who undergo intensive training (e.g., Chandy, 2019; The Star, 2021). To address this conflict, policymakers have initiated a long‐term plan focused on public education for prevention purposes and the implementation of suitable interventions (Ministry of Youth and Sports, n.d.). The incorrect conceptualization of gaming disorder could have adverse consequences in various areas (Aarseth et al., 2017; Ji et al., 2022). Empirical investigations also yield conflicting findings, outlining both the detrimental (e.g., Lin et al., 2019) and adaptive aspects of gaming (e.g., Halbrook et al., 2019). Recognizing the need to safeguard the welfare of esports players, this research was conducted to explore the conceptual link between excessive gaming and psychological distress.

Empirically, esports players demonstrate significantly higher motivation in gaming compared to their regular gaming counterparts (Bányai et al., 2019a, 2019b, 2020; Martončik, 2015). This heightened involvement increases their vulnerability to psychiatric concerns (Bányai et al., 2019a, 2019b, 2020). Notably, empirical findings indicate that the relationship between IGD and psychiatric concerns is indistinguishable between esports and regular players (Bányai et al., 2019a, 2019b, 2020). This finding might seem counterintuitive since esports players, who spend more time gaming than regular players, would be expected to have a higher risk of developing IGD. However, a possible explanation is that the duration of gaming alone is not a reliable indicator of pathological gaming (Király et al., 2017; Vuorre et al., 2022). Alternatively, it can be interpreted that prolonged gaming, both for esports players and regular gamers, diminishes the capacity for a healthy lifestyle, rendering them both susceptible to poor health outcomes (Chan et al., 2022). Consequently, policymakers must gain a comprehensive understanding of the primary stakeholders in this flourishing industry. This research specifically targets esports players, who are integral to the industry's success. Examining the underlying theoretical rationales for IGD, this research aligns with the call for further investigation in this area (APA, 2013) to better serve the esports community (Bányai et al., 2019a, 2019b).

The Internet Gaming Disorder Scale‐Short Form (IGDS9‐SF; Pontes & Griffiths, 2015) serves as a measure for evaluating the severity of the tentative diagnosis of IGD outlined by the APA (2013). The proposed criteria for IGD include saliency, withdrawal, tolerance, relapse, conflict, and problematic outcomes, mirroring those for the Gaming Addiction Scale (GAS; Lemmens et al., 2009). Additionally, IGD criteria include the loss of interest in previous hobbies and a tendency to conceal the amount of gaming. The interchangeability of the terms “excessive gaming” and “internet gaming disorder” in empirical studies (Jain & Jain, 2021; Lin et al., 2019; Monacis et al., 2016; Rapinda et al., 2021; Severo et al., 2020; Wong et al., 2020; Yen et al., 2019) has blurred the conceptual differences between these two indicators of problematic gaming. Both excessive gaming and IGD are empirically linked to psychological distress. To prevent the pathologization of normal behavior (Brunborg et al., 2013, 2015; Charlton & Danforth, 2007), further research on the tentative diagnosis of IGD is crucial (APA, 2013). This research can help clarify distinctions and better inform the community and relevant stakeholders.

While empirical evidence consistently links IGD to various indicators of psychological distress (González‐Bueso et al., 2018), it is crucial to approach these findings with a critical perspective. Notably, a relevant study found a significant link between IGD and stress, but no significant association with depression and anxiety (Jaafar et al., 2021). These inconsistencies underscore the ongoing challenge of interpreting the cumulative findings and highlight the need to address conflicting views regarding the validity of IGD as a clinical concern (e.g., Aarseth et al., 2017). Recent attempts to understand IGD suggest that it may result from certain vulnerabilities (Rapinda et al., 2021), addressing identified issues in the literature, such as the lack of consolidated theories and longitudinal designs (Rosendo‐Rios et al., 2022). These limitations are tied to concerns about the directionality of the findings. Without a clear theoretical perspective, interpreting the proposed conceptual relationship between IGD and associated clinical concerns becomes challenging. IGD, previously linked to mental health issues (González‐Bueso et al., 2018), might either be the cause or the outcome of these concerns. The findings are inconsistent, as demonstrated in research by Jaafar et al. (2021), and longitudinal studies have not provided a clear direction for these effects. For instance, while excessive gaming heightens the risk of clinical issues, the vulnerabilities stemming from excessive gaming do not appear to increase susceptibility to further problematic gaming (e.g., Chang et al., 2022). This contradicts previous findings regarding predispositions as vulnerability factors for IGD (e.g., Chen et al., 2021). The absence of a strong theoretical framework adds complexity to this issue, as it fails to provide a clear direction for the proposed connections between gaming and psychological outcomes. Therefore, the current literature needs further development to consider the possibility that IGD might evolve into an obsolete diagnosis (Aarseth et al., 2017; Ji et al., 2022).

Recent developments suggest that the GAS (Lemmens et al., 2009) is best represented by a two‐factor model (Brunborg et al., 2015), highlighting the intricate nature of the conceptual construct of addictive gaming. These two factors, identified as core and peripheral criteria for gaming addiction (Charlton, 2002), shed light on the nuances of intense gaming. The peripheral criteria, encompassing high saliency, mood modification, and tolerance, reflect the intensity of gaming (Charlton, 2002). However, the impact of intense gaming remains unclear. Recent findings propose that feelings of loneliness and psychological distress drive players to invest more in gaming (André et al., 2020), suggesting the possibility of adaptive gaming. Without a clear indication of causality, it is plausible to interpret excessive gaming as a result or outcome of experienced psychological distress, consistent with many empirical findings on the maladaptive aspects of excessive gaming (e.g., Chan et al., 2022). Nonetheless, existing sources indicate the possibility that this form of gaming does not necessarily cause complications for players but may instead be adaptive, aiding them in coping with negative psychological experiences (Brunborg et al., 2013; Charlton & Danforth, 2007; Vuorre et al., 2022). The addition of core criteria—withdrawal, relapse, conflict, and problem (Brunborg et al., 2013, 2015; Charlton & Danforth, 2007)—to the existing indicators of intense gaming escalates active gaming to gaming addiction. Theoretically, healthy players often engage in the peripheral characteristics (saliency, mood modification, and tolerance), while pathological players frequently exhibit the core characteristics (Brunborg et al., 2013, 2015; Charlton & Danforth, 2007). Thus, estimated scores for peripheral features can indicate engagement levels, while scores for core features reflect the degree of gaming addiction (Brunborg et al., 2015). Recent findings suggest that core characteristics better reflect underlying addictive tendencies, while peripheral symptoms are context‐dependent (Snodgrass et al., 2019). The former core characteristics are grounded in neurobiological processes, while the latter peripheral characteristics involve learned patterns of thoughts and practices common in a given context (Snodgrass et al., 2019). From this perspective, both core and peripheral features increase susceptibility to addictive gaming, and the neurological processes of gaming are linked to expectations about gaming, expressed symptoms, and unique contextual experiences (Snodgrass et al., 2019).

Fundamentally, gaming can be viewed as a form of adaptive coping (e.g., André et al., 2020), challenging the prevailing idea that extended gaming is inherently maladaptive (e.g., Chan et al., 2022) and opposing interpretations that link it directly to IGD (e.g., Jain & Jain, 2021). This shift in conceptualizing gaming behavior highlights its intricate nature and the complexities embedded within proposed theoretical frameworks. Oversimplifying the outcomes of these constructs may introduce bias, influencing interpretations of their implications, particularly in the rapidly expanding gaming industry. Moreover, disregarding the potential positive aspects of gaming risks unfairly labeling normal gaming behavior as pathological (Brunborg et al., 2013, 2015; Charlton & Danforth, 2007).

Presently, there is uncertainty surrounding the defined conceptual terms of IGD and the core as well as peripheral characteristics associated with gaming. As previously mentioned, these attributes could be viewed as individual differences that potentially heighten susceptibility to IGD (e.g., Snodgrass et al., 2019). Hence, the prominence of these features plays a pivotal role in determining the prominence of IGD. Considering these characteristics as factors that augment vulnerability to IGD seems a more rational approach rather than assuming their equivalence. This overlap raises concerns about “jingle‐jangle” fallacies (Marsh, 1994). The “jingle” fallacy involves falsely assuming resemblance between two distinct theoretical constructs (Thorndike, 1904), while the “jangle” fallacy entails falsely presuming distinctness between two similar theoretical constructs (Kelley, 1927). These fallacies pose significant concerns as they cloud the interpretation of the represented theoretical constructs (Gaylord‐Harden et al., 2010; Lee, 2021; Paulhus et al., 2004; Watson et al., 2013). To address this overlap, this research proposes a solution: employing statistical controls to manage confounding variables.

A confounder serves as a variable that distorts the relationship between two other variables (e.g., MacKinnon et al., 2000). Essentially, it obscures or weakens the direct impact of a predictor on an outcome. A basic confounder's conceptual model resembles a simple mediation model but with a reversed pathway from the predictor to the designated mediator (MacKinnon et al., 2000). Wysocki et al. (2022) have outlined several conceptual models for confounders, with two pertinent ones being the confounder and the collider. The collider model suggests a direct effect of the predictor on both the confounder and the outcome, as well as an effect of the outcome on the confounder, but this has less relevance in the current context. The confounder model, akin to MacKinnon et al.'s (2000) description, is more pertinent here. It proposes direct effects of the confounder on the predictor and the outcome, along with a direct effect of the predictor on the outcome. Crucially, the confounder predates the predictor and has links to the outcome (MacKinnon et al., 2000). Considering this, the core and peripheral characteristics described by Snodgrass et al. (2019) seem to fulfill the criteria of being confounders to the direct effect of IGD on indicators of psychological distress. Therefore, the significant association observed between IGD and psychological distress indicators (e.g., Rapinda et al., 2021) might potentially mask the effects of these characteristics. Consequently, prematurely classifying IGD as a disorder could undermine understanding the true origins of the adverse effects of unregulated gaming.

This study endeavors to build upon current literature, which primarily delves into illustrating the impact of gaming aspects on psychological outcomes (e.g., Chen, Chen, Gamble, et al., 2022). It seeks to prolong the longitudinal perspective by shedding light on how the overlap of these gaming aspects might cloud their connection with psychological outcomes. By utilizing longitudinal data from a sample of esports players in Malaysia—an underrepresented demographic in current research—this paper aims to examine whether the core and peripheral characteristics identified by Snodgrass et al. (2019) distorted the direct relationship between IGD and indicators of psychological distress. Apart from its academic significance, this research is pertinent for industry stakeholders, including esports players, and governing bodies.

## METHOD

### Participants

This longitudinal study involved adults (aged 18 years and above) in Malaysia who are active esports players, having taken part in at least one official esports tournament. A group of 100 esports players in Malaysia volunteered to participate in this longitudinal research, comprising data collected at two distinct time points. Table 1 provides an overview of the demographic characteristics of these participants.

**TABLE 1:** Participants' demographics.

Demographic	*N* (%)
Age	
18–30	80 (80%)
31–43	17 (17%)
44–56	3 (3%)
Gender	
Male	95% (95)
Female	5 (5%)
Employment status	
Full time	52 (52%)
Part time	12 (12%)
Unemployed	36 (36%)
Marital status	
Married	18 (18%)
Single	82 (82%)
Education level	
Primary education	1 (1%)
Secondary education	17 (17%)
Tertiary education	80 (80%)
Not relevant	2 (2%)
Monthly income	
RM 500–RM 1000	34 (34%)
RM 1001–RM 2000	15 (15%)
RM 2001–RM 3000	16 (16%)
RM 3001–RM 4000	13 (13%)
RM 4001–RM 5000	4 (4%)
RM 5001 and above	12 (12%)
Unwilling to disclose	6 (6%)
Ethnicity	
Malay	70 (70%)
Chinese	16 (16%)
Indian	5 (5%)
Non‐Muslim natives	6 (6%)
Muslim natives	3 (3%)

Abbreviation: RM, Ringgit Malaysia (currency used in Malaysia).

### Procedure

The initial data collection phase took place over the course of 1 month, starting from 3 April 2022, and concluding on 31 May 2022. The study's recruitment was facilitated through the survey company's website, where individuals interested and meeting specific criteria (Malaysian citizenship, age 18 and above, and participation in at least one official esports tournament) were invited to participate in the survey. These participants have given their consent to take part in this research, which includes subsequent follow‐up survey. Following a three‐month interval, the same cohort of 100 esports players was contacted for a follow‐up survey, which occurred between 31 August 31, and 31 October 2022. It is worth emphasizing that this longitudinal research effectively retained the participation of these esports players for the follow‐up survey. As a token of appreciation for their involvement, each participant received RM10 (~$2.28), upon completing each phase of data collection. Furthermore, it is important to underscore that the research received ethical approval from Monash University Human Ethics Committee (Project ID: 31775), ensuring that it adhered to established ethical research guidelines and standards.

### Statistical analysis

Statistical analysis was conducted with multilevel generalized structured component analysis (GSCA; see Hwang et al., 2007). This choice of analysis considered how the observed effects may differ across different timeframes by allowing both loadings and path coefficients to vary across time. Hence, this analysis portrays the changes in the examined relationships that occur over time. GSCA involves three sub‐models, including the measurement, structural, and weighted relation models. The measurement model describes the relationships between the components and their respective indicators, the structural model specifies the relationships between the components, and the weighted relation model defines components as weighted sums of their indicators. This procedure incorporates these sub‐models into a single model. This statistical method provides details on model fit, such as the explanatory power of components and indicators (e.g., FIT and AFIT). The GSCA models posited disordered gaming as the predictor of the indicators of psychological distress, while the core and peripheral components were posited as the intervening variables. Empirical findings indicate that this statistical method has no convergence issue with small samples (Jung et al., 2018). To determine the statistical significance of the parameter estimates, the critical ratios (the parameter estimates divided by their standard error) were used to determine the significance of the parameter estimates (Hwang et al., 2007). A parameter estimate is assumed to be statistically significant at 0.05 alpha level if the critical ratio is greater than 2 (Hwang et al., 2007). The effect size of the estimated pathways was indicated by *f* ^2^, in which the values of 0.02, 0.15, and 0.35 were considered small, medium, and large, respectively (Cohen, 1988).

Four GSCA models were estimated for each indicator of psychological distress. Three of these models depict the direct effect of the gaming aspects on the indicators of psychological distress, and the last model depicts the confounding effects of the core and peripheral characteristics on the direct effect of IGD and the indicator of psychological distress. The rationale behind isolating these outcomes is to provide a clear and distinct demonstration of the confounding impact on each specific indicator of psychological distress.

### Measures

The respondents' demographic details, encompassing age, gender, and ethnicity, were gathered for descriptive analysis purposes.

Gaming addiction was measured using the GAS composed by Lemmens et al. (2009). This scale comprises seven items, such as asking participants about thoughts like spending the entire day playing games over the last 6 months. Respondents rated these items on a five‐point scale, ranging from 1 (*never*) to 5 (*very often*). The initial three items contributed to forming the core construct, while the remaining four items contributed to defining the peripheral construct, aligning with the structure outlined by Brunborg et al. (2015).

The measurement of gaming disorder utilized the IGDS9‐SF (Pontes & Griffiths, 2015), which includes nine items. For example: “Have you experienced increased irritability, anxiety, or sadness when attempting to cut down or stop gaming?” Participants rated these items on a scale of 1 (*never*) to 5 (*very often*).

The Depression, Anxiety, and Stress Scale 21 (DASS‐21; Lovibond & Lovibond, 1995) was used to assess depression, anxiety, and stress. This scale comprises 21 items, with seven items each reflecting the respective subscales for depression (e.g., “I couldn't seem to experience any positive feeling at all”), anxiety (e.g., “I felt I was close to panic”), and stress (e.g., “I found it hard to wind down”). Participants rated these items on a four‐point scale ranging from 0 (*Did not apply to me at all*) to 3 (*Applied to me very much or most of the time*).

## RESULTS

The first step involved examining the confounding models, where all weights and item loadings were found to be statistically significant, barring two items within the IGDS9‐SF. These two items were removed, and the significance persisted across the remaining items. Subsequent models were then constructed to explore the direct influence of IGD and its core and peripheral characteristics on indicators of psychological distress. Further details, including reliability estimates and the estimated weights and loadings, are presented in the Supporting Information.

**FIGURE 1:**
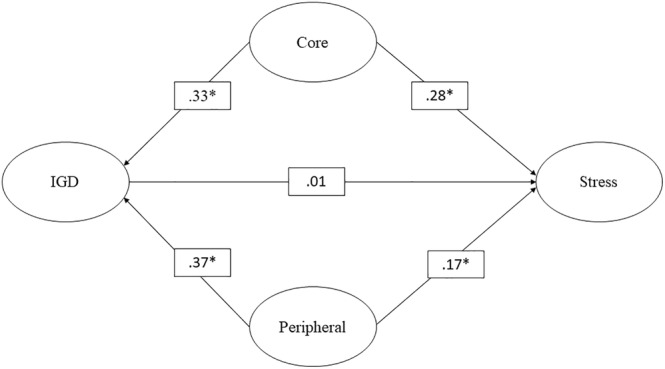
Confounding model in predicting stress. *significant; IGD, internet gaming disorder.

Table 2 provides an overview of the outcomes from the GSCA models concerning stress as the outcome variable. The average estimated direct impact of IGD on stress was found to be statistically significant. Additionally, the non‐significance of the random effect suggests a consistent estimated direct effect over a 3‐month time span. Similarly, the direct effects of both core and peripheral characteristics on stress were significant. The estimated random effects also lacked significance, indicating consistent effects across the observed time interval. Despite IGD losing significance in predicting stress according to the confounding model, the core and peripheral characteristics maintained their predictive significance. Notably, the core characteristic emerged as the most influential predictor for stress. Furthermore, the estimated random effects reaffirmed the consistency of these effects throughout the research timeframe. Figure 1 illustrates the confounding model's structure with stress as the outcome variable.

**TABLE 2:** Summary of the GSCA models predicting stress.

Path (*f* ^*2*^)	Between			Random		
	estimate	SE	CR	estimate	SE	CR
Model 1a (FIT = 0.31)						
IGD → Stress (0.04)	0.20	0.07	2.90	0.08	0.12	0.67
Model 2a (FIT = 0.38)						
Peripheral → Stress (0.07)	0.27	0.08	3.31	0.13	0.15	0.85
Model 3a (FIT = 0.37)						
Core → Stress (0.12)	0.33	0.07	4.53	0.13	0.15	0.86
Model 4a (FIT = 0.34)						
Peripheral → IGD (0.16)	0.37	0.07	5.13	0.04	0.09	0.41
Core → IGD (0.12)	0.33	0.09	3.71	0.07	0.12	0.62
IGD → Stress (0.00)	0.01	0.11	0.09	0.01	0.13	0.10
Peripheral → Stress (0.03)	0.17	0.10	1.80	0.11	0.16	0.73
Core → Stress (0.08)	0.28	0.09	2.96	0.12	0.16	0.75

Abbreviations: CR, critical ratio; GSCA, generalized structured component analysis; IGD, internet gaming disorder.

**FIGURE 2:**
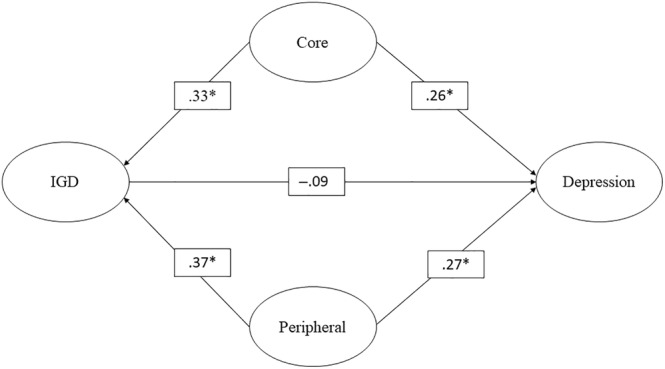
Confounding model in predicting depression. *significant; IGD, internet gaming disorder.

Table 3 summarizes the findings derived from the GSCA models focusing on depression as the outcome. The average estimated direct effect of IGD on depression did not reach statistical significance. However, both the core and peripheral characteristics exhibited significant direct effects. Furthermore, the estimated random effects did not show significance, suggesting consistent direct effects across the observed time span. In the confounding model, a noticeable shift appeared in the IGD–depression relationship, previously positive but now depicted as negative, although this alteration lacked statistical significance. Conversely, the positive direct impacts of core and peripheral characteristics on depression remained statistically significant. Additionally, the estimated random effects validated the consistent nature of these impacts throughout the study period. Figure 2 illustrates the structure of the confounding model concerning depression as the outcome variable.

**TABLE 3:** Summary of the GSCA models predicting depression.

Path (*f* ^*2*^)	Between			Random		
	estimate	SE	CR	estimate	SE	CR
Model 1b (FIT = 0.33)						
IGD → Depression (0.02)	0.13	0.09	1.46	0.01	0.10	0.13
Model 2b (FIT = 0.41)						
Peripheral → Depression (0.10)	0.30	0.08	3.95	0.09	0.13	0.66
Model 3b (FIT = 0.39)						
Core → Depression (0.12)	0.33	0.08	4.37	0.14	0.16	0.86
Model 4b (FIT = 0.35)						
Peripheral → IGD (0.15)	0.37	0.07	5.12	0.04	0.10	0.40
Core → IGD (0.12)	0.33	0.09	3.66	0.07	0.12	0.61
IGD → Depression (0.01)	−0.09	0.12	0.76	0.07	0.17	0.45
Peripheral → Depression (0.08)	0.27	0.10	2.61	0.09	0.15	0.58
Core → Depression (0.07)	0.26	0.09	2.74	0.19	0.20	0.96

Abbreviations: CR, critical ratio; GSCA, generalized structured component analysis; IGD, internet gaming disorder.

**FIGURE 3:**
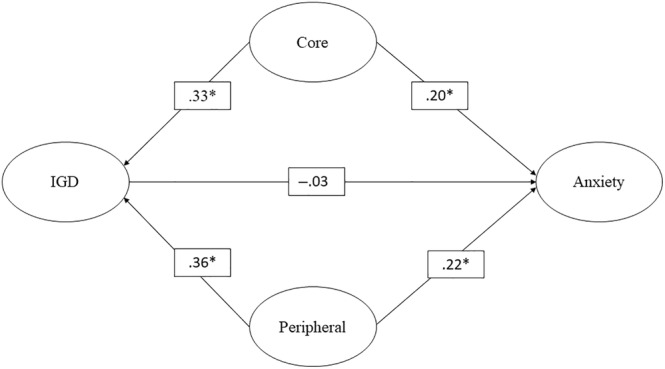
Confounding model in predicting anxiety. *significant; IGD, internet gaming disorder.

Table 4 presents the findings from the GSCA models focused on anxiety as the outcome variable. Similar to depression and stress, the direct impact of IGD on anxiety did not yield statistical significance. Conversely, the direct effects of both core and peripheral characteristics were statistically significant. Moreover, the estimated random effects demonstrated consistency across the observed time interval, lacking significance. Within the confounding model, an apparent alteration emerged in the IGD–anxiety relationship, previously positive but now depicted as negative, although this change did not achieve statistical significance. Nonetheless, the positive direct effects of core and peripheral characteristics on anxiety remained significant. Furthermore, the estimated random effects affirmed the consistency of these effects across the research period. Figure 3 illustrates the structure of the confounding model concerning anxiety as the outcome variable.

**TABLE 4:** Summary of the GSCA models predicting anxiety.

Path (*f* ^*2*^)	Between			Random		
	estimate	SE	CR	estimate	SE	CR
Model 1c (FIT = 0.31)						
IGD → Anxiety (0.02)	0.13	0.08	1.62	0.08	0.12	0.61
Model 2c (FIT = 0.37)						
Peripheral → Anxiety (0.07)	0.25	0.08	3.24	0.10	0.13	0.72
Model 3c (FIT = 0.36)						
Core → Anxiety (0.06)	0.25	0.08	3.18	0.16	0.17	0.97
Model 4c (FIT = 0.35)						
Peripheral → IGD (0.15)	0.36	0.07	5.10	0.04	0.10	0.40
Core → IGD (0.13)	0.33	0.09	3.62	0.07	0.12	0.61
IGD → Anxiety (0.00)	−0.03	0.12	0.22	0.07	0.17	0.45
Peripheral → Anxiety (0.05)	0.22	0.10	2.15	0.09	0.15	0.58
Core → Anxiety (0.03)	0.2	0.1	2.00	0.19	0.20	0.96

Abbreviations: CR, critical ratio; GSCA, generalized structured component analysis; IGD, internet gaming disorder.

## DISCUSSION

This paper investigated whether the core and peripheral characteristics outlined by Snodgrass et al. (2019) have influenced the direct impact of IGD on indicators of psychological distress. Therefore, this focus represents a significant expansion of the current literature, which predominantly explores the impact of these gaming aspects on psychological outcomes (e.g., Chen, Chen, Gamble, et al., 2022). By delving into distinct aspects of psychological distress, this research extends prior studies on the correlation between intense gaming and psychological distress (Bányai et al., 2019a, 2019b, 2020). It also addresses conceptual gaps by examining theories with longitudinal designs (Rosendo‐Rios et al., 2022).

This longitudinal study aims to address concerns by untangling the conceptual differences between gaming disorder and fundamental gaming features in predicting various indicators of psychological distress. The findings indicate the confounding effects of core and peripheral characteristics on the direct relationship between IGD and stress. Similarly, these characteristics confound the direct relationship between IGD and depression as well as anxiety, altering the direction of these direct effects. The longitudinal design offers diverse perspectives by showcasing the stability of the confounding model and the direct effects over time.

The results supported the connection between IGD and stress, aligning with prior research (Wong et al., 2020; Yen et al., 2019), indicating a link between IGD and heightened stress levels among esports players. However, the obtained findings suggest that the direct impact of IGD on stress is overshadowed by the influence of the core and peripheral characteristics outlined by Snodgrass et al. (2019). With these inherent traits considered, IGD itself appears inconsequential in stress development. The findings suggest that an underlying neurobiological process (core characteristics), contributing to susceptibility to prolonged and unregulated gaming, plays a pivotal role in stress development. While context‐specific vulnerabilities (peripheral characteristics) hold significance, their influence seems to be lesser compared to neurobiological predispositions.

The results diverged from prior findings (Wong et al., 2020; Yen et al., 2019) by not supporting a connection between IGD and depression, suggesting that unregulated, prolonged gaming does not inherently trigger emotional distress in esports players. Interestingly, depression seems to play a pivotal role in shaping the severity of IGD; when statistically accounting for depression, IGD loses its significant predictive power for stress (Yen et al., 2019). Hence, a plausible interpretation emerges: problematic gaming is predominantly linked to depressive states (Chang et al., 2022), aligning with the emotional regulation aspect of IGD (APA, 2013). The contradictory finding can be explained by the type of sample used in this research. This study focused on competitive, professional esports players, differing from participants in prior research (e.g., Yen et al., 2019). These esports players might possess enhanced control over critical psychological processes relevant to sports performance, such as precompetitive emotional states (Brevers et al., 2020). Hence, despite their prolonged gaming, they may exhibit lower tendencies toward negative emotions. This distinction between esports and regular players contrasts with previous findings (Bányai et al., 2019a, 2019b, 2020). The absence of regular players in this research points to the potential for future studies to compare esports and regular players, offering significant insights. However, both core and peripheral characteristics significantly predicted depression, indicating that existing vulnerabilities in unregulated gaming heighten susceptibility to negative emotions among esports players. While the measured IGD did not directly correlate with depression, these predispositions might have manifested differently in gaming aspects or other observable behaviors leading to depression.

The findings did not establish a significant link between IGD and anxiety among esports players, suggesting that extended, unregulated gaming did not evoke anxiety in this group. This outcome mirrors the absence of a significant relationship found between IGD and depression in this study. This might be explained by the high comorbidity observed between depression and anxiety (e.g., Naranjo et al., 2019), indicating a shared underlying cause for these symptoms (e.g., Baldwin et al., 2002). This could also be related to esports players' mastery over critical psychological processes relevant to sports performance (Brevers et al., 2020). Moreover, both core and peripheral characteristics significantly predicted anxiety, indicating that existing vulnerabilities to gaming addiction heightened susceptibility to anxiety among esports players. This finding aligns with the conceptual links between these traits and anxiety. However, these traits did not manifest through IGD. It is plausible that the core and peripheral characteristics might surface in different aspects of gaming or in observable behaviors triggering anxiety.

This research tackled the concern of the jingle‐jangle fallacy. While IGD indeed correlates significantly with traits that elevate susceptibility to addictive gaming, the results of the confounding analysis suggest that these conceptual terms might operate independently. For instance, in the analysis focusing on stress as the outcome, the traits associated with addictive gaming diminished the significance of IGD's direct effect on stress. This indicates that once these traits are accounted for, IGD loses its predictive power regarding stress—a hint at the conceptual relationship between these traits and IGD. As previously mentioned, the core and peripheral characteristics represent inherent predispositions that heighten the risk of IGD (e.g., Snodgrass et al., 2019). This suggests that the presence of these traits determines the relevance of IGD. Therefore, theoretically, these aspects need to exist before IGD can develop. Furthermore, the impacts of the core and peripheral traits on depression and anxiety appear to function independently from IGD. These outcomes also suggest the redundancy of IGD when considering these existing traits. Thus, assuming IGD and these fundamental traits are equivalent would amount to committing the jingle fallacy—an erroneous assumption about the similarity of two distinct theoretical constructs (Thorndike, 1904).

The adaptability of intense gaming to players' psychological well‐being lacks evidence, contrasting earlier research findings (André et al., 2020; Johannes et al., 2021). Regarding the effects on depression, anxiety, and stress, the peripheral characteristics are better interpreted as context‐dependent vulnerabilities to unregulated gaming (Snodgrass et al., 2019). Consequently, these findings contradict the previous notion that peripheral characteristics signify gaming engagement (Brunborg et al., 2013, 2015; Charlton & Danforth, 2007). Previously, the prominence of core features suggested an intensified form of intense gaming or peripheral features (Charlton, 2002; Charlton & Danforth, 2007). However, the results indicate a need to reassess the conceptual relationship between core and peripheral features, as the latter may not represent leisure gaming or pleasure derived from gaming (Charlton, 2002). Instead, these findings propose interpreting these features as individual differences signifying vulnerability to problematic gaming (Snodgrass et al., 2019). Consequently, an esports player could develop IGD suddenly without the necessity for escalated intense gaming. Moreover, these findings extend to show that these aspects are associated with indicators of psychological distress. Hence, this research offers deeper insights into core and peripheral characteristics, unlike previous studies that merely described these constructs without exploring their conceptual relationships with other psychological variables (e.g., Snodgrass et al., 2019). This research significantly contributes to the ongoing debate regarding the diagnosis of IGD (APA, 2013). Based on the current literature, endorsing IGD as a diagnosable condition would be premature. The measured IGD did not significantly predict indicators of psychological distress, suggesting that IGD itself does not impair the functioning of current competitive esports players. Instead, it is the existing inclinations (e.g., core and peripheral features; Snodgrass et al., 2019) that relate to distress aspects. While vulnerabilities are evident, prior research also suggests the existence of innate features that might act protectively against IGD (e.g., Brevers et al., 2020). This aligns with the recommendation for further efforts to substantiate the decision to formalize IGD as a diagnosable condition (APA, 2013).

The moderate effect sizes observed suggest that additional psychological variables might underpin the direct relationships between gaming aspects and psychological distress indicators. For instance, the demanding lifestyle of competitive esports players could intersect with a proclivity for excessive gaming, potentially compromising their psychological well‐being (Bányai et al., 2019a, 2019b). Likewise, inherent deficits, such as lacking characteristics associated with good sportsmanship, might intensify the link between gaming and psychological distress (Brevers et al., 2020). Within this context, the identified core and peripheral features might have triggered other vulnerabilities, heightening esports players' susceptibility to depression, stress, and anxiety. The intrinsic characteristics of these core and peripheral features at the individual level suggest that specific conditions or environmental factors can stimulate their emergence. This aligns with the previous idea that specific contexts or traits within the sample might impact the results. This underscores the importance of further validating the decision to classify IGD as a diagnosable condition (APA, 2013).

This study has several limitations. Firstly, its longitudinal design involved only one follow‐up survey, restricting the generated findings to the period under investigation while the esports players continued their active gaming involvement. This indicates the necessity for ongoing investigations to broaden the scope of understanding. Secondly, the analysis flagged concerns about the psychometrics of the utilized measurements. There is a need to validate these measures within a Malaysian sample, especially among the current cohort of esports players, which could significantly contribute to the existing literature. This also implies the necessity to reevaluate the conceptual constructs of GA and IGD, potentially warranting a reconsideration of psychological measurement methods. Additionally, the research suffered from a small sample size, constrained by practical limitations. Unlike previous studies (e.g., *N_esports_* = 204; Bányai et al., 2019a, 2019b), the stringent inclusion criteria led to a limited sample size of 100 respondents. Relaxing these criteria might increase the sample size but could introduce more variability, potentially compromising the validity and reliability of the findings.

The study concluded that the core and peripheral characteristics were significant predictors of IGD severity, supporting their role as vulnerabilities in unregulated, excessive gaming. This suggests that esports players exhibiting prominent core and peripheral characteristics are at a higher risk of developing IGD. The analysis highlighted that these characteristics influenced the connection between IGD and stress. Once these features were considered, IGD no longer held a significant association with the stress levels of esports players. Additionally, the core and peripheral traits notably predicted depression and anxiety. Given the lack of significant direct effects of IGD on depression and anxiety, the concern regarding IGD may be premature.

## CONFLICT OF INTEREST STATEMENT

The authors have no conflicts of interest to declare.

## ETHICS STATEMENT

Ethics approval was granted by the Monash University Human Ethics Committee (Project ID: 31775).

## Supporting information


**Data S1.** Supporting information.
