# Supplementary material for: Confounding analysis with gaming aspects in predicting psychological distress of esports players
Source: Psych J. 2024 Feb 1;13(4):541–51. doi: 10.1002/pchj.728 (PMC11317182; doi:10.1002/pchj.728)
Supplement: Supplementary file 1 — Data S1. Supporting information. [file PCHJ-13-541-s001.docx]

Weights and Loadings for Model 1a

|  | Total | | | | | | | | | Baseline | | | Three months | | |
| --- | --- | --- | --- | --- | --- | --- | --- | --- | --- | --- | --- | --- | --- | --- | --- |
|  | Weight (Between) | | | Loading (Between) | | | Loading (Random) | | | Loading | | | Loading | | |
|  | Estimate | S.E. | C.R. | Estimate | S.E. | C.R. | Estimate | S.E. | C.R. | Estimate | S.E. | C.R. | Estimate | S.E. | C.R. |
| *Internet gaming disorder (ρ = .71)* |  |  |  |  |  |  |  |  |  |  |  |  |  |  |  |
| IGD2 | 0.35 | 0.06 | 5.86 | 0.65 | 0.08 | 7.83 | 0.01 | 0.09 | 0.06 | 0.66 | 0.12 | 5.27 | 0.65 | 0.1 | 6.35 |
| IGD3 | 0.25 | 0.07 | 3.6 | 0.47 | 0.12 | 3.98 | 0.02 | 0.09 | 0.18 | 0.45 | 0.13 | 3.37 | 0.49 | 0.15 | 3.37 |
| IGD4 | 0.33 | 0.06 | 6.02 | 0.62 | 0.08 | 7.36 | 0.09 | 0.12 | 0.75 | 0.52 | 0.11 | 4.56 | 0.71 | 0.11 | 6.75 |
| IGD5 | 0.24 | 0.07 | 3.47 | 0.44 | 0.12 | 3.55 | 0.01 | 0.11 | 0.13 | 0.45 | 0.15 | 3.06 | 0.43 | 0.16 | 2.72 |
| IGD6 | 0.29 | 0.06 | 5.01 | 0.55 | 0.1 | 5.57 | 0.01 | 0.08 | 0.07 | 0.55 | 0.13 | 4.36 | 0.56 | 0.11 | 4.86 |
| IGD7 | 0.25 | 0.07 | 3.68 | 0.44 | 0.11 | 4.18 | 0.12 | 0.17 | 0.69 | 0.56 | 0.16 | 3.58 | 0.32 | 0.15 | 2.08 |
| IGD9 | 0.19 | 0.08 | 2.59 | 0.37 | 0.13 | 2.77 | 0.01 | 0.12 | 0.07 | 0.38 | 0.15 | 2.49 | 0.36 | 0.18 | 1.99 |
| Stress *(ρ = .84)* |  |  |  |  |  |  |  |  |  |  |  |  |  |  |  |
| S1 | 0.23 | 0.03 | 8.68 | 0.68 | 0.68 | 0.05 | 0.12 | 0.12 | 0.99 | 0.81 | 0.07 | 12.32 | 0.56 | 0.09 | 6.44 |
| S2 | 0.18 | 0.03 | 6.67 | 0.57 | 0.56 | 0.07 | 0.04 | 0.09 | 0.42 | 0.6 | 0.08 | 7.48 | 0.53 | 0.11 | 4.69 |
| S3 | 0.21 | 0.02 | 8.51 | 0.66 | 0.65 | 0.06 | 0.03 | 0.07 | 0.37 | 0.68 | 0.07 | 9.17 | 0.63 | 0.09 | 7.09 |
| S4 | 0.26 | 0.03 | 8.19 | 0.77 | 0.78 | 0.05 | 0.11 | 0.14 | 0.84 | 0.67 | 0.1 | 6.65 | 0.9 | 0.05 | 16.49 |
| S5 | 0.24 | 0.04 | 6.51 | 0.70 | 0.71 | 0.06 | 0.08 | 0.09 | 0.86 | 0.63 | 0.08 | 7.56 | 0.79 | 0.08 | 10.38 |
| S6 | 0.16 | 0.03 | 5.94 | 0.52 | 0.5 | 0.08 | 0.03 | 0.08 | 0.38 | 0.54 | 0.08 | 6.59 | 0.47 | 0.11 | 4.19 |
| S7 | 0.23 | 0.02 | 10.28 | 0.70 | 0.7 | 0.04 | 0.01 | 0.07 | 0.17 | 0.71 | 0.07 | 10.41 | 0.69 | 0.07 | 9.36 |

Weights and Loadings for Model 2a

|  | Total | | | | | | | | | Baseline | | | Three months | | |
| --- | --- | --- | --- | --- | --- | --- | --- | --- | --- | --- | --- | --- | --- | --- | --- |
|  | Weight (Between) | | | Loading (Between) | | | Loading (Random) | | | Loading | | | Loading | | |
|  | Estimate | S.E. | C.R. | Estimate | S.E. | C.R. | Estimate | S.E. | C.R. | Estimate | S.E. | C.R. | Estimate | S.E. | C.R. |
| Peripheral *(ρ = .75)* |  |  |  |  |  |  |  |  |  |  |  |  |  |  |  |
| GA1 | 0.49 | 0.05 | 9.72 | 0.69 | 0.07 | 10.53 | 0.09 | 0.09 | 0.93 | 0.60 | 0.10 | 6.26 | 0.77 | 0.06 | 13.44 |
| GA2 | 0.50 | 0.05 | 9.40 | 0.71 | 0.07 | 10.85 | 0.02 | 0.05 | 0.32 | 0.69 | 0.07 | 9.86 | 0.72 | 0.08 | 8.96 |
| GA3 | 0.47 | 0.07 | 7.22 | 0.66 | 0.09 | 7.47 | 0.11 | 0.11 | 0.95 | 0.77 | 0.09 | 8.09 | 0.55 | 0.11 | 4.96 |
| Stress *(ρ = .84)* |  |  |  |  |  |  |  |  |  |  |  |  |  |  |  |
| S1 | 0.22 | 0.03 | 8.83 | 0.68 | 0.05 | 13.73 | 0.12 | 0.12 | 1.00 | 0.80 | 0.07 | 12.15 | 0.56 | 0.08 | 6.66 |
| S2 | 0.18 | 0.03 | 6.76 | 0.56 | 0.07 | 7.92 | 0.04 | 0.09 | 0.43 | 0.60 | 0.08 | 7.61 | 0.53 | 0.11 | 4.78 |
| S3 | 0.21 | 0.02 | 8.49 | 0.65 | 0.06 | 10.39 | 0.03 | 0.07 | 0.37 | 0.68 | 0.08 | 8.93 | 0.63 | 0.09 | 7.08 |
| S4 | 0.26 | 0.03 | 8.05 | 0.78 | 0.05 | 17.11 | 0.11 | 0.14 | 0.82 | 0.67 | 0.10 | 6.55 | 0.90 | 0.06 | 16.17 |
| S5 | 0.24 | 0.04 | 6.16 | 0.71 | 0.06 | 11.27 | 0.08 | 0.09 | 0.86 | 0.63 | 0.09 | 7.34 | 0.79 | 0.08 | 10.27 |
| S6 | 0.17 | 0.03 | 6.18 | 0.51 | 0.08 | 6.60 | 0.03 | 0.08 | 0.36 | 0.54 | 0.08 | 6.63 | 0.48 | 0.11 | 4.20 |
| S7 | 0.22 | 0.02 | 11.34 | 0.70 | 0.04 | 16.20 | 0.01 | 0.07 | 0.18 | 0.71 | 0.07 | 10.41 | 0.68 | 0.07 | 9.63 |

Weights and Loadings for Model 3a

|  | Total | | | | | | | | | Baseline | | | Three months | | |
| --- | --- | --- | --- | --- | --- | --- | --- | --- | --- | --- | --- | --- | --- | --- | --- |
|  | Weight (Between) | | | Loading (Between) | | | Loading (Random) | | | Loading | | | Loading | | |
|  | Estimate | S.E. | C.R. | Estimate | S.E. | C.R. | Estimate | S.E. | C.R. | Estimate | S.E. | C.R. | Estimate | S.E. | C.R. |
| Core *(ρ = .69)* |  |  |  |  |  |  |  |  |  |  |  |  |  |  |  |
| GA4 | 0.26 | 0.09 | 2.80 | 0.41 | 0.16 | 2.61 | 0.11 | 0.14 | 0.84 | 0.66 | 0.10 | 6.48 | 0.88 | 0.06 | 15.15 |
| GA5 | 0.22 | 0.10 | 2.34 | 0.37 | 0.16 | 2.37 | 0.08 | 0.09 | 0.86 | 0.62 | 0.09 | 7.27 | 0.78 | 0.08 | 9.96 |
| GA6 | 0.48 | 0.05 | 9.37 | 0.77 | 0.06 | 13.03 | 0.03 | 0.08 | 0.32 | 0.54 | 0.08 | 7.00 | 0.49 | 0.11 | 4.49 |
| GA7 | 0.54 | 0.06 | 9.47 | 0.81 | 0.04 | 18.41 | 0.01 | 0.07 | 0.11 | 0.71 | 0.07 | 10.33 | 0.70 | 0.07 | 9.65 |
| Stress *(ρ = .84)* |  |  |  |  |  |  |  |  |  |  |  |  |  |  |  |
| S1 | 0.23 | 0.02 | 9.08 | 0.68 | 0.05 | 13.76 | 0.07 | 0.12 | 0.55 | 0.48 | 0.20 | 2.44 | 0.34 | 0.17 | 2.02 |
| S2 | 0.19 | 0.03 | 7.11 | 0.57 | 0.07 | 7.97 | 0.10 | 0.14 | 0.72 | 0.27 | 0.19 | 1.42 | 0.47 | 0.17 | 2.80 |
| S3 | 0.21 | 0.02 | 8.51 | 0.66 | 0.06 | 11.01 | 0.06 | 0.11 | 0.52 | 0.82 | 0.08 | 10.34 | 0.71 | 0.10 | 6.95 |
| S4 | 0.24 | 0.03 | 7.46 | 0.77 | 0.05 | 16.77 | 0.04 | 0.08 | 0.51 | 0.77 | 0.06 | 12.01 | 0.85 | 0.08 | 11.32 |
| S5 | 0.23 | 0.04 | 6.41 | 0.70 | 0.06 | 10.88 | 0.12 | 0.12 | 1.02 | 0.80 | 0.06 | 12.44 | 0.56 | 0.08 | 6.66 |
| S6 | 0.18 | 0.03 | 6.40 | 0.52 | 0.07 | 6.99 | 0.03 | 0.09 | 0.38 | 0.61 | 0.08 | 7.54 | 0.54 | 0.11 | 4.84 |
| S7 | 0.23 | 0.02 | 10.28 | 0.70 | 0.04 | 15.95 | 0.02 | 0.07 | 0.31 | 0.68 | 0.07 | 9.43 | 0.64 | 0.09 | 7.37 |

Weights and Loadings for Model 4a

|  | Total | | | | | | | | | Baseline | | | Three months | | |
| --- | --- | --- | --- | --- | --- | --- | --- | --- | --- | --- | --- | --- | --- | --- | --- |
|  | Weight (Between) | | | Loading (Between) | | | Loading (Random) | | | Loading | | | Loading | | |
|  | Estimate | S.E. | C.R. | Estimate | S.E. | C.R. | Estimate | S.E. | C.R. | Estimate | S.E. | C.R. | Estimate | S.E. | C.R. |
| *Internet gaming disorder (ρ = .71)* |  |  |  |  |  |  |  |  |  |  |  |  |  |  |  |
| IGD2 | 0.31 | 0.06 | 5.46 | 0.62 | 0.08 | 7.92 | 0.00 | 0.09 | 0.03 | 0.62 | 0.12 | 5.01 | 0.62 | 0.09 | 6.74 |
| IGD3 | 0.27 | 0.06 | 4.34 | 0.49 | 0.11 | 4.62 | 0.01 | 0.09 | 0.15 | 0.48 | 0.12 | 3.91 | 0.50 | 0.13 | 3.77 |
| IGD4 | 0.33 | 0.05 | 6.01 | 0.60 | 0.08 | 7.53 | 0.09 | 0.12 | 0.74 | 0.52 | 0.11 | 4.90 | 0.69 | 0.10 | 6.74 |
| IGD5 | 0.29 | 0.06 | 4.41 | 0.48 | 0.12 | 4.19 | 0.02 | 0.11 | 0.22 | 0.51 | 0.13 | 3.75 | 0.46 | 0.15 | 3.06 |
| IGD6 | 0.33 | 0.05 | 6.25 | 0.58 | 0.09 | 6.54 | 0.00 | 0.08 | 0.06 | 0.59 | 0.11 | 5.14 | 0.58 | 0.10 | 5.65 |
| IGD7 | 0.20 | 0.06 | 3.11 | 0.39 | 0.10 | 3.86 | 0.11 | 0.17 | 0.68 | 0.51 | 0.16 | 3.24 | 0.28 | 0.14 | 1.98 |
| IGD9 | 0.20 | 0.08 | 2.47 | 0.37 | 0.13 | 2.82 | 0.01 | 0.11 | 0.08 | 0.38 | 0.14 | 2.73 | 0.36 | 0.18 | 1.97 |
| Peripheral *(ρ = .72)* |  |  |  |  |  |  |  |  |  |  |  |  |  |  |  |
| GA1 | 0.43 | 0.05 | 7.98 | 0.64 | 0.07 | 8.83 | 0.09 | 0.10 | 0.96 | 0.55 | 0.10 | 5.35 | 0.74 | 0.07 | 11.13 |
| GA2 | 0.56 | 0.06 | 9.18 | 0.75 | 0.06 | 11.91 | 0.02 | 0.05 | 0.39 | 0.73 | 0.07 | 10.52 | 0.77 | 0.08 | 10.13 |
| GA3 | 0.46 | 0.07 | 6.75 | 0.65 | 0.09 | 7.20 | 0.11 | 0.11 | 0.95 | 0.76 | 0.10 | 7.84 | 0.54 | 0.11 | 4.76 |
| Core (ρ = .70) |  |  |  |  |  |  |  |  |  |  |  |  |  |  |  |
| GA4 | 0.25 | 0.09 | 2.64 | 0.40 | 0.16 | 2.59 | 0.11 | 0.14 | 0.84 | 0.66 | 0.10 | 6.40 | 0.89 | 0.06 | 15.73 |
| GA5 | 0.32 | 0.10 | 3.12 | 0.45 | 0.15 | 3.01 | 0.08 | 0.09 | 0.85 | 0.63 | 0.09 | 7.23 | 0.78 | 0.08 | 10.18 |
| GA6 | 0.49 | 0.06 | 8.85 | 0.76 | 0.06 | 12.53 | 0.03 | 0.08 | 0.33 | 0.54 | 0.08 | 6.91 | 0.49 | 0.11 | 4.58 |
| GA7 | 0.50 | 0.06 | 8.34 | 0.77 | 0.06 | 12.73 | 0.01 | 0.07 | 0.11 | 0.71 | 0.07 | 10.36 | 0.69 | 0.07 | 9.46 |
| Stress *(ρ = .84)* |  |  |  |  |  |  |  |  |  |  |  |  |  |  |  |
| S1 | 0.23 | 0.03 | 8.46 | 0.68 | 0.05 | 13.50 | 0.07 | 0.12 | 0.53 | 0.47 | 0.20 | 2.40 | 0.34 | 0.17 | 2.01 |
| S2 | 0.18 | 0.03 | 6.55 | 0.57 | 0.07 | 7.89 | 0.08 | 0.13 | 0.63 | 0.37 | 0.19 | 1.95 | 0.54 | 0.16 | 3.43 |
| S3 | 0.22 | 0.03 | 8.18 | 0.66 | 0.06 | 10.95 | 0.06 | 0.11 | 0.52 | 0.82 | 0.09 | 9.46 | 0.71 | 0.10 | 7.27 |
| S4 | 0.23 | 0.03 | 7.07 | 0.77 | 0.05 | 16.67 | 0.03 | 0.08 | 0.41 | 0.74 | 0.08 | 9.78 | 0.81 | 0.09 | 9.00 |
| S5 | 0.24 | 0.04 | 6.12 | 0.70 | 0.06 | 10.98 | 0.12 | 0.12 | 1.01 | 0.80 | 0.07 | 12.30 | 0.56 | 0.08 | 6.60 |
| S6 | 0.18 | 0.03 | 6.47 | 0.52 | 0.07 | 7.03 | 0.04 | 0.09 | 0.40 | 0.60 | 0.08 | 7.46 | 0.53 | 0.11 | 4.84 |
| S7 | 0.23 | 0.02 | 9.40 | 0.70 | 0.04 | 15.97 | 0.02 | 0.07 | 0.31 | 0.68 | 0.07 | 9.52 | 0.64 | 0.09 | 7.29 |

Weights and Loadings for Model 1b

|  | Total | | | | | | | | | Baseline | | | Three months | | |
| --- | --- | --- | --- | --- | --- | --- | --- | --- | --- | --- | --- | --- | --- | --- | --- |
|  | Weight (Between) | | | Loading (Between) | | | Loading (Random) | | | Loading | | | Loading | | |
|  | Estimate | S.E. | C.R. | Estimate | S.E. | C.R. | Estimate | S.E. | C.R. | Estimate | S.E. | C.R. | Estimate | S.E. | C.R. |
| *Internet gaming disorder (ρ = .70)* |  |  |  |  |  |  |  |  |  |  |  |  |  |  |  |
| IGD2 | 0.36 | 0.06 | 5.79 | 0.66 | 0.09 | 7.59 | 0.00 | 0.09 | 0.04 | 0.66 | 0.13 | 5.20 | 0.65 | 0.10 | 6.25 |
| IGD3 | 0.26 | 0.07 | 3.73 | 0.48 | 0.12 | 4.04 | 0.02 | 0.09 | 0.20 | 0.46 | 0.14 | 3.42 | 0.50 | 0.14 | 3.45 |
| IGD4 | 0.32 | 0.06 | 5.43 | 0.61 | 0.09 | 6.87 | 0.09 | 0.13 | 0.72 | 0.52 | 0.12 | 4.39 | 0.70 | 0.11 | 6.33 |
| IGD5 | 0.25 | 0.08 | 3.29 | 0.45 | 0.13 | 3.37 | 0.02 | 0.11 | 0.15 | 0.46 | 0.16 | 2.99 | 0.43 | 0.16 | 2.62 |
| IGD6 | 0.29 | 0.06 | 4.94 | 0.55 | 0.10 | 5.48 | 0.01 | 0.08 | 0.07 | 0.54 | 0.13 | 4.24 | 0.56 | 0.12 | 4.82 |
| IGD7 | 0.23 | 0.07 | 3.22 | 0.43 | 0.11 | 3.77 | 0.12 | 0.17 | 0.71 | 0.55 | 0.16 | 3.38 | 0.30 | 0.15 | 1.97 |
| IGD9 | 0.21 | 0.08 | 2.54 | 0.38 | 0.14 | 2.72 | 0.01 | 0.12 | 0.06 | 0.38 | 0.16 | 2.46 | 0.37 | 0.19 | 1.98 |
| Depression *(ρ = .86)* |  |  |  |  |  |  |  |  |  |  |  |  |  |  |  |
| D1 | 0.18 | 0.02 | 8.28 | 0.56 | 0.07 | 8.06 | 0.11 | 0.12 | 0.97 | 0.44 | 0.09 | 4.97 | 0.67 | 0.09 | 7.20 |
| D2 | 0.19 | 0.02 | 11.06 | 0.64 | 0.05 | 12.15 | 0.07 | 0.08 | 0.86 | 0.71 | 0.08 | 8.69 | 0.57 | 0.06 | 9.56 |
| D3 | 0.17 | 0.03 | 6.56 | 0.59 | 0.08 | 7.56 | 0.01 | 0.08 | 0.17 | 0.58 | 0.12 | 4.79 | 0.60 | 0.07 | 8.08 |
| D4 | 0.23 | 0.02 | 12.00 | 0.76 | 0.04 | 18.59 | 0.02 | 0.06 | 0.29 | 0.77 | 0.06 | 12.19 | 0.74 | 0.06 | 12.41 |
| D5 | 0.24 | 0.02 | 11.94 | 0.80 | 0.04 | 22.63 | 0.01 | 0.05 | 0.12 | 0.80 | 0.06 | 13.77 | 0.79 | 0.05 | 14.89 |
| D6 | 0.21 | 0.02 | 10.11 | 0.71 | 0.05 | 14.84 | 0.01 | 0.06 | 0.21 | 0.73 | 0.07 | 10.58 | 0.70 | 0.07 | 9.90 |
| D7 | 0.22 | 0.02 | 10.74 | 0.74 | 0.04 | 17.05 | 0.00 | 0.06 | 0.02 | 0.74 | 0.08 | 9.84 | 0.74 | 0.06 | 12.95 |

Weights and Loadings for Model 2b

|  | Total | | | | | | | | | Baseline | | | Three months | | |
| --- | --- | --- | --- | --- | --- | --- | --- | --- | --- | --- | --- | --- | --- | --- | --- |
|  | Weight (Between) | | | Loading (Between) | | | Loading (Random) | | | Loading | | | Loading | | |
|  | Estimate | S.E. | C.R. | Estimate | S.E. | C.R. | Estimate | S.E. | C.R. | Estimate | S.E. | C.R. | Estimate | S.E. | C.R. |
| Peripheral *(ρ = .72)* |  |  |  |  |  |  |  |  |  |  |  |  |  |  |  |
| GA1 | 0.49 | 0.05 | 9.93 | 0.69 | 0.06 | 10.77 | 0.09 | 0.09 | 0.97 | 0.59 | 0.09 | 6.50 | 0.78 | 0.06 | 13.11 |
| GA2 | 0.46 | 0.06 | 7.50 | 0.68 | 0.08 | 8.79 | 0.02 | 0.05 | 0.31 | 0.66 | 0.08 | 8.46 | 0.69 | 0.10 | 7.20 |
| GA3 | 0.51 | 0.06 | 7.95 | 0.69 | 0.08 | 8.51 | 0.10 | 0.11 | 0.93 | 0.79 | 0.08 | 9.88 | 0.59 | 0.11 | 5.34 |
| Depression *(ρ = .86)* |  |  |  |  |  |  |  |  |  |  |  |  |  |  |  |
| D1 | 0.17 | 0.02 | 8.26 | 0.55 | 0.07 | 7.92 | 0.12 | 0.12 | 0.96 | 0.43 | 0.09 | 4.72 | 0.66 | 0.09 | 7.23 |
| D2 | 0.20 | 0.02 | 10.40 | 0.64 | 0.05 | 12.27 | 0.07 | 0.08 | 0.86 | 0.72 | 0.08 | 8.70 | 0.57 | 0.06 | 9.60 |
| D3 | 0.16 | 0.03 | 5.61 | 0.58 | 0.08 | 7.17 | 0.02 | 0.08 | 0.24 | 0.56 | 0.12 | 4.53 | 0.60 | 0.08 | 7.74 |
| D4 | 0.22 | 0.02 | 11.45 | 0.75 | 0.04 | 18.27 | 0.01 | 0.06 | 0.25 | 0.77 | 0.07 | 11.65 | 0.74 | 0.06 | 12.63 |
| D5 | 0.23 | 0.02 | 10.89 | 0.79 | 0.04 | 20.92 | 0.00 | 0.05 | 0.04 | 0.79 | 0.06 | 12.88 | 0.79 | 0.05 | 14.50 |
| D6 | 0.24 | 0.02 | 11.38 | 0.73 | 0.04 | 16.97 | 0.02 | 0.06 | 0.25 | 0.75 | 0.07 | 11.34 | 0.72 | 0.07 | 10.52 |
| D7 | 0.22 | 0.02 | 9.86 | 0.75 | 0.04 | 17.44 | 0.00 | 0.06 | 0.03 | 0.75 | 0.07 | 10.15 | 0.74 | 0.06 | 13.08 |

Weights and Loadings for Model 3b

|  | Total | | | | | | | | | Baseline | | | Three months | | |
| --- | --- | --- | --- | --- | --- | --- | --- | --- | --- | --- | --- | --- | --- | --- | --- |
|  | Weight (Between) | | | Loading (Between) | | | Loading (Random) | | | Loading | | | Loading | | |
|  | Estimate | S.E. | C.R. | Estimate | S.E. | C.R. | Estimate | S.E. | C.R. | Estimate | S.E. | C.R. | Estimate | S.E. | C.R. |
| Core (ρ = .70) |  |  |  |  |  |  |  |  |  |  |  |  |  |  |  |
| GA4 | 0.26 | 0.09 | 2.79 | 0.41 | 0.16 | 2.58 | 0.02 | 0.06 | 0.27 | 0.77 | 0.06 | 12.25 | 0.74 | 0.06 | 12.18 |
| GA5 | 0.20 | 0.10 | 1.89 | 0.34 | 0.17 | 2.07 | 0.00 | 0.05 | 0.07 | 0.80 | 0.06 | 13.62 | 0.79 | 0.05 | 14.86 |
| GA6 | 0.47 | 0.05 | 9.38 | 0.76 | 0.06 | 12.38 | 0.01 | 0.06 | 0.14 | 0.71 | 0.07 | 9.85 | 0.70 | 0.07 | 10.07 |
| GA7 | 0.57 | 0.06 | 9.26 | 0.82 | 0.04 | 18.41 | 0.00 | 0.06 | 0.00 | 0.74 | 0.08 | 9.83 | 0.74 | 0.06 | 12.85 |
| Depression *(ρ = .84)* |  |  |  |  |  |  |  |  |  |  |  |  |  |  |  |
| D1 | 0.18 | 0.02 | 8.54 | 0.56 | 0.07 | 8.26 | 0.07 | 0.13 | 0.56 | 0.48 | 0.20 | 2.42 | 0.34 | 0.17 | 1.99 |
| D2 | 0.22 | 0.02 | 10.28 | 0.66 | 0.05 | 12.84 | 0.10 | 0.14 | 0.73 | 0.24 | 0.20 | 1.23 | 0.44 | 0.18 | 2.50 |
| D3 | 0.16 | 0.03 | 5.54 | 0.58 | 0.08 | 7.26 | 0.06 | 0.11 | 0.52 | 0.82 | 0.08 | 10.06 | 0.70 | 0.11 | 6.69 |
| D4 | 0.22 | 0.02 | 11.31 | 0.75 | 0.04 | 18.62 | 0.05 | 0.09 | 0.55 | 0.78 | 0.06 | 12.68 | 0.87 | 0.08 | 11.14 |
| D5 | 0.24 | 0.02 | 11.46 | 0.80 | 0.04 | 22.35 | 0.11 | 0.12 | 0.97 | 0.45 | 0.09 | 4.84 | 0.67 | 0.09 | 7.63 |
| D6 | 0.19 | 0.02 | 9.78 | 0.71 | 0.05 | 13.88 | 0.08 | 0.09 | 0.91 | 0.74 | 0.08 | 9.30 | 0.58 | 0.06 | 9.70 |
| D7 | 0.23 | 0.02 | 10.76 | 0.74 | 0.04 | 16.62 | 0.02 | 0.09 | 0.21 | 0.56 | 0.13 | 4.49 | 0.60 | 0.08 | 8.01 |

Weights and Loadings for Model 4b

|  | Total | | | | | | | | | Baseline | | | Three months | | |
| --- | --- | --- | --- | --- | --- | --- | --- | --- | --- | --- | --- | --- | --- | --- | --- |
|  | Weight (Between) | | | Loading (Between) | | | Loading (Random) | | | Loading | | | Loading | | |
|  | Estimate | S.E. | C.R. | Estimate | S.E. | C.R. | Estimate | S.E. | C.R. | Estimate | S.E. | C.R. | Estimate | S.E. | C.R. |
| *Internet gaming disorder (ρ = .71)* |  |  |  |  |  |  |  |  |  |  |  |  |  |  |  |
| IGD2 | 0.31 | 0.05 | 5.68 | 0.62 | 0.08 | 7.91 | 0.00 | 0.09 | 0.04 | 0.62 | 0.12 | 4.93 | 0.62 | 0.09 | 6.76 |
| IGD3 | 0.28 | 0.06 | 4.38 | 0.50 | 0.10 | 4.74 | 0.01 | 0.09 | 0.16 | 0.48 | 0.12 | 4.05 | 0.51 | 0.13 | 3.82 |
| IGD4 | 0.32 | 0.06 | 5.72 | 0.60 | 0.08 | 7.30 | 0.09 | 0.11 | 0.76 | 0.51 | 0.11 | 4.82 | 0.69 | 0.10 | 6.67 |
| IGD5 | 0.28 | 0.07 | 4.25 | 0.48 | 0.12 | 4.08 | 0.02 | 0.10 | 0.22 | 0.51 | 0.14 | 3.68 | 0.46 | 0.15 | 2.99 |
| IGD6 | 0.33 | 0.05 | 6.36 | 0.59 | 0.09 | 6.74 | 0.01 | 0.08 | 0.07 | 0.59 | 0.11 | 5.28 | 0.58 | 0.10 | 5.69 |
| IGD7 | 0.20 | 0.06 | 3.10 | 0.39 | 0.10 | 3.77 | 0.11 | 0.17 | 0.69 | 0.51 | 0.16 | 3.20 | 0.28 | 0.14 | 2.02 |
| IGD9 | 0.19 | 0.08 | 2.34 | 0.36 | 0.13 | 2.77 | 0.01 | 0.11 | 0.07 | 0.37 | 0.14 | 2.66 | 0.35 | 0.18 | 1.94 |
| Peripheral *(ρ = .72)* |  |  |  |  |  |  |  |  |  |  |  |  |  |  |  |
| GA1 | 0.43 | 0.05 | 8.08 | 0.64 | 0.07 | 8.67 | 0.10 | 0.10 | 0.98 | 0.54 | 0.10 | 5.27 | 0.74 | 0.07 | 10.89 |
| GA2 | 0.53 | 0.06 | 8.15 | 0.72 | 0.07 | 10.75 | 0.02 | 0.05 | 0.42 | 0.70 | 0.07 | 9.69 | 0.74 | 0.08 | 9.17 |
| GA3 | 0.50 | 0.07 | 7.04 | 0.68 | 0.09 | 7.76 | 0.11 | 0.11 | 0.95 | 0.79 | 0.09 | 8.82 | 0.58 | 0.11 | 5.06 |
| Core (ρ = .70) |  |  |  |  |  |  |  |  |  |  |  |  |  |  |  |
| GA4 | 0.24 | 0.09 | 2.61 | 0.40 | 0.15 | 2.60 | 0.01 | 0.06 | 0.24 | 0.77 | 0.06 | 11.82 | 0.74 | 0.06 | 12.15 |
| GA5 | 0.32 | 0.11 | 3.02 | 0.45 | 0.15 | 2.96 | 0.00 | 0.06 | 0.01 | 0.79 | 0.06 | 12.50 | 0.79 | 0.05 | 14.72 |
| GA6 | 0.49 | 0.05 | 9.67 | 0.76 | 0.06 | 12.13 | 0.01 | 0.06 | 0.18 | 0.73 | 0.07 | 10.42 | 0.71 | 0.07 | 10.04 |
| GA7 | 0.49 | 0.06 | 7.84 | 0.77 | 0.06 | 12.41 | 0.00 | 0.06 | 0.01 | 0.75 | 0.07 | 9.99 | 0.75 | 0.06 | 12.81 |
| Depression *(ρ = .84)* |  |  |  |  |  |  |  |  |  |  |  |  |  |  |  |
| D1 | 0.17 | 0.02 | 7.42 | 0.55 | 0.07 | 7.77 | 0.06 | 0.12 | 0.53 | 0.46 | 0.19 | 2.40 | 0.34 | 0.17 | 2.02 |
| D2 | 0.22 | 0.02 | 9.79 | 0.66 | 0.05 | 13.06 | 0.08 | 0.13 | 0.64 | 0.37 | 0.19 | 1.95 | 0.54 | 0.16 | 3.34 |
| D3 | 0.15 | 0.03 | 4.93 | 0.57 | 0.08 | 7.05 | 0.06 | 0.11 | 0.53 | 0.82 | 0.09 | 9.63 | 0.71 | 0.10 | 7.16 |
| D4 | 0.22 | 0.02 | 10.65 | 0.75 | 0.04 | 18.67 | 0.03 | 0.08 | 0.42 | 0.74 | 0.07 | 10.07 | 0.81 | 0.09 | 8.74 |
| D5 | 0.23 | 0.02 | 9.89 | 0.79 | 0.04 | 20.61 | 0.12 | 0.12 | 0.97 | 0.44 | 0.09 | 4.62 | 0.67 | 0.09 | 7.17 |
| D6 | 0.21 | 0.02 | 9.11 | 0.72 | 0.05 | 14.80 | 0.08 | 0.09 | 0.89 | 0.73 | 0.08 | 9.19 | 0.58 | 0.06 | 9.86 |
| D7 | 0.23 | 0.03 | 9.33 | 0.75 | 0.04 | 17.18 | 0.02 | 0.09 | 0.27 | 0.55 | 0.13 | 4.28 | 0.60 | 0.08 | 7.83 |

Weights and Loadings for Model 1c

|  | Total | | | | | | | | | Baseline | | | Three months | | |
| --- | --- | --- | --- | --- | --- | --- | --- | --- | --- | --- | --- | --- | --- | --- | --- |
|  | Weight (Between) | | | Loading (Between) | | | Loading (Random) | | | Loading | | | Loading | | |
|  | Estimate | S.E. | C.R. | Estimate | S.E. | C.R. | Estimate | S.E. | C.R. | Estimate | S.E. | C.R. | Estimate | S.E. | C.R. |
| *Internet gaming disorder (ρ = .71)* |  |  |  |  |  |  |  |  |  |  |  |  |  |  |  |
| IGD2 | 0.35 | 0.06 | 5.46 | 0.65 | 0.09 | 7.31 | 0.00 | 0.10 | 0.04 | 0.65 | 0.13 | 4.99 | 0.64 | 0.11 | 6.09 |
| IGD3 | 0.26 | 0.07 | 3.92 | 0.48 | 0.12 | 4.15 | 0.02 | 0.09 | 0.19 | 0.46 | 0.13 | 3.53 | 0.50 | 0.14 | 3.54 |
| IGD4 | 0.33 | 0.06 | 5.51 | 0.62 | 0.09 | 6.88 | 0.09 | 0.12 | 0.74 | 0.53 | 0.12 | 4.52 | 0.71 | 0.11 | 6.31 |
| IGD5 | 0.25 | 0.07 | 3.43 | 0.44 | 0.13 | 3.48 | 0.02 | 0.11 | 0.15 | 0.46 | 0.15 | 3.03 | 0.43 | 0.16 | 2.68 |
| IGD6 | 0.29 | 0.06 | 4.86 | 0.55 | 0.10 | 5.35 | 0.00 | 0.08 | 0.06 | 0.55 | 0.13 | 4.22 | 0.56 | 0.12 | 4.79 |
| IGD7 | 0.24 | 0.07 | 3.16 | 0.43 | 0.12 | 3.69 | 0.12 | 0.17 | 0.71 | 0.55 | 0.16 | 3.36 | 0.31 | 0.16 | 1.98 |
| IGD9 | 0.20 | 0.08 | 2.55 | 0.37 | 0.14 | 2.72 | 0.01 | 0.12 | 0.07 | 0.38 | 0.16 | 2.46 | 0.37 | 0.18 | 1.98 |
| Anxiety *(ρ = .83)* |  |  |  |  |  |  |  |  |  |  |  |  |  |  |  |
| A1 | 0.17 | 0.03 | 5.31 | 0.51 | 0.08 | 6.24 | 0.02 | 0.08 | 0.19 | 0.53 | 0.12 | 4.54 | 0.50 | 0.09 | 5.40 |
| A2 | 0.17 | 0.03 | 5.07 | 0.50 | 0.08 | 6.10 | 0.10 | 0.18 | 0.55 | 0.40 | 0.15 | 2.64 | 0.60 | 0.14 | 4.36 |
| A3 | 0.25 | 0.02 | 11.01 | 0.72 | 0.05 | 15.49 | 0.07 | 0.10 | 0.68 | 0.78 | 0.08 | 9.44 | 0.65 | 0.07 | 9.92 |
| A4 | 0.26 | 0.02 | 12.75 | 0.78 | 0.04 | 21.87 | 0.01 | 0.07 | 0.20 | 0.79 | 0.07 | 11.58 | 0.77 | 0.06 | 13.79 |
| A5 | 0.25 | 0.03 | 9.26 | 0.74 | 0.05 | 14.64 | 0.03 | 0.10 | 0.35 | 0.77 | 0.09 | 8.88 | 0.70 | 0.09 | 7.90 |
| A6 | 0.17 | 0.03 | 6.28 | 0.51 | 0.08 | 6.46 | 0.03 | 0.08 | 0.45 | 0.54 | 0.09 | 5.73 | 0.48 | 0.10 | 4.99 |
| A7 | 0.24 | 0.02 | 11.47 | 0.73 | 0.05 | 15.67 | 0.08 | 0.10 | 0.78 | 0.65 | 0.09 | 7.35 | 0.82 | 0.06 | 14.74 |

Weights and Loadings for Model 2c

|  | Total | | | | | | | | | Baseline | | | Three months | | |
| --- | --- | --- | --- | --- | --- | --- | --- | --- | --- | --- | --- | --- | --- | --- | --- |
|  | Weight (Between) | | | Loading (Between) | | | Loading (Random) | | | Loading | | | Loading | | |
|  | Estimate | S.E. | C.R. | Estimate | S.E. | C.R. | Estimate | S.E. | C.R. | Estimate | S.E. | C.R. | Estimate | S.E. | C.R. |
| Peripheral *(ρ = .72)* |  |  |  |  |  |  |  |  |  |  |  |  |  |  |  |
| GA1 | 0.48 | 0.05 | 8.77 | 0.68 | 0.07 | 9.78 | 0.09 | 0.10 | 0.96 | 0.59 | 0.10 | 5.98 | 0.77 | 0.06 | 12.16 |
| GA2 | 0.48 | 0.06 | 8.34 | 0.69 | 0.07 | 9.59 | 0.02 | 0.05 | 0.32 | 0.67 | 0.07 | 9.10 | 0.71 | 0.09 | 7.82 |
| GA3 | 0.50 | 0.07 | 7.49 | 0.68 | 0.08 | 8.08 | 0.10 | 0.11 | 0.94 | 0.79 | 0.09 | 9.21 | 0.58 | 0.11 | 5.19 |
| Anxiety *(ρ = .83)* |  |  |  |  |  |  |  |  |  |  |  |  |  |  |  |
| A1 | 0.16 | 0.03 | 5.75 | 0.51 | 0.08 | 6.51 | 0.01 | 0.08 | 0.18 | 0.52 | 0.11 | 4.66 | 0.49 | 0.09 | 5.48 |
| A2 | 0.17 | 0.03 | 4.75 | 0.50 | 0.08 | 5.97 | 0.10 | 0.18 | 0.55 | 0.40 | 0.15 | 2.62 | 0.59 | 0.14 | 4.34 |
| A3 | 0.23 | 0.02 | 9.96 | 0.71 | 0.05 | 14.88 | 0.06 | 0.10 | 0.67 | 0.77 | 0.08 | 9.10 | 0.64 | 0.07 | 9.60 |
| A4 | 0.27 | 0.02 | 11.82 | 0.79 | 0.04 | 22.25 | 0.02 | 0.07 | 0.25 | 0.80 | 0.07 | 11.72 | 0.77 | 0.06 | 13.99 |
| A5 | 0.25 | 0.02 | 10.11 | 0.74 | 0.05 | 15.61 | 0.04 | 0.10 | 0.37 | 0.77 | 0.08 | 9.38 | 0.70 | 0.09 | 7.89 |
| A6 | 0.17 | 0.02 | 7.05 | 0.51 | 0.07 | 6.82 | 0.03 | 0.07 | 0.44 | 0.54 | 0.09 | 5.95 | 0.47 | 0.09 | 5.18 |
| A7 | 0.26 | 0.02 | 12.23 | 0.74 | 0.05 | 15.73 | 0.08 | 0.10 | 0.78 | 0.66 | 0.09 | 7.53 | 0.82 | 0.06 | 14.76 |

Weights and Loadings for Model 3c

|  | Total | | | | | | | | | Baseline | | | Three months | | |
| --- | --- | --- | --- | --- | --- | --- | --- | --- | --- | --- | --- | --- | --- | --- | --- |
|  | Weight (Between) | | | Loading (Between) | | | Loading (Random) | | | Loading | | | Loading | | |
|  | Estimate | S.E. | C.R. | Estimate | S.E. | C.R. | Estimate | S.E. | C.R. | Estimate | S.E. | C.R. | Estimate | S.E. | C.R. |
| Core (ρ = .70) |  |  |  |  |  |  |  |  |  |  |  |  |  |  |  |
| GA4 | 0.29 | 0.10 | 2.81 | 0.44 | 0.17 | 2.60 | 0.07 | 0.12 | 0.54 | 0.51 | 0.21 | 2.43 | 0.37 | 0.18 | 2.10 |
| GA5 | 0.21 | 0.11 | 1.90 | 0.35 | 0.17 | 2.08 | 0.11 | 0.14 | 0.74 | 0.25 | 0.20 | 1.23 | 0.46 | 0.18 | 2.56 |
| GA6 | 0.49 | 0.05 | 10.06 | 0.77 | 0.06 | 12.89 | 0.05 | 0.11 | 0.49 | 0.82 | 0.08 | 10.29 | 0.72 | 0.10 | 7.10 |
| GA7 | 0.52 | 0.06 | 9.28 | 0.80 | 0.05 | 16.60 | 0.05 | 0.09 | 0.52 | 0.75 | 0.06 | 11.74 | 0.84 | 0.08 | 10.38 |
| Anxiety *(ρ = .83)* |  |  |  |  |  |  |  |  |  |  |  |  |  |  |  |
| A1 | 0.17 | 0.03 | 5.65 | 0.52 | 0.08 | 6.52 | 0.02 | 0.08 | 0.22 | 0.54 | 0.11 | 4.68 | 0.50 | 0.09 | 5.51 |
| A2 | 0.18 | 0.03 | 5.92 | 0.51 | 0.08 | 6.21 | 0.10 | 0.18 | 0.55 | 0.41 | 0.16 | 2.59 | 0.61 | 0.13 | 4.76 |
| A3 | 0.26 | 0.02 | 10.38 | 0.72 | 0.04 | 16.26 | 0.07 | 0.10 | 0.69 | 0.79 | 0.08 | 9.50 | 0.65 | 0.06 | 10.14 |
| A4 | 0.24 | 0.02 | 11.18 | 0.77 | 0.04 | 20.55 | 0.01 | 0.07 | 0.17 | 0.79 | 0.07 | 10.66 | 0.76 | 0.06 | 13.81 |
| A5 | 0.25 | 0.03 | 9.84 | 0.73 | 0.05 | 15.06 | 0.03 | 0.09 | 0.35 | 0.76 | 0.09 | 8.90 | 0.70 | 0.09 | 7.88 |
| A6 | 0.17 | 0.02 | 6.74 | 0.51 | 0.08 | 6.65 | 0.04 | 0.08 | 0.46 | 0.54 | 0.09 | 5.80 | 0.47 | 0.09 | 5.08 |
| A7 | 0.24 | 0.02 | 11.78 | 0.73 | 0.05 | 16.13 | 0.08 | 0.11 | 0.76 | 0.65 | 0.09 | 7.53 | 0.81 | 0.06 | 14.49 |

Weights and Loadings for Model 4c

|  | Total | | | | | | | | | Baseline | | | Three months | | |
| --- | --- | --- | --- | --- | --- | --- | --- | --- | --- | --- | --- | --- | --- | --- | --- |
|  | Weight (Between) | | | Loading (Between) | | | Loading (Random) | | | Loading | | | Loading | | |
|  | Estimate | S.E. | C.R. | Estimate | S.E. | C.R. | Estimate | S.E. | C.R. | Estimate | S.E. | C.R. | Estimate | S.E. | C.R. |
| *Internet gaming disorder (ρ = .71)* |  |  |  |  |  |  |  |  |  |  |  |  |  |  |  |
| IGD2 | 0.31 | 0.06 | 5.30 | 0.62 | 0.08 | 7.47 | 0.00 | 0.09 | 0.03 | 0.62 | 0.13 | 4.75 | 0.62 | 0.10 | 6.44 |
| IGD3 | 0.27 | 0.06 | 4.38 | 0.49 | 0.11 | 4.65 | 0.01 | 0.09 | 0.14 | 0.48 | 0.12 | 3.89 | 0.50 | 0.13 | 3.81 |
| IGD4 | 0.33 | 0.06 | 5.57 | 0.60 | 0.08 | 7.29 | 0.09 | 0.12 | 0.73 | 0.52 | 0.11 | 4.76 | 0.69 | 0.10 | 6.57 |
| IGD5 | 0.29 | 0.07 | 4.27 | 0.48 | 0.12 | 4.11 | 0.02 | 0.11 | 0.22 | 0.51 | 0.14 | 3.73 | 0.46 | 0.15 | 3.01 |
| IGD6 | 0.33 | 0.05 | 6.33 | 0.59 | 0.09 | 6.66 | 0.01 | 0.08 | 0.07 | 0.59 | 0.11 | 5.21 | 0.58 | 0.10 | 5.61 |
| IGD7 | 0.20 | 0.07 | 2.93 | 0.39 | 0.11 | 3.66 | 0.11 | 0.17 | 0.68 | 0.51 | 0.16 | 3.17 | 0.28 | 0.15 | 1.89 |
| IGD9 | 0.20 | 0.08 | 2.40 | 0.37 | 0.13 | 2.78 | 0.01 | 0.12 | 0.08 | 0.38 | 0.14 | 2.65 | 0.36 | 0.18 | 1.96 |
| Peripheral *(ρ = .72)* |  |  |  |  |  |  |  |  |  |  |  |  |  |  |  |
| GA1 | 0.42 | 0.06 | 7.50 | 0.64 | 0.08 | 8.33 | 0.10 | 0.10 | 0.97 | 0.54 | 0.11 | 5.07 | 0.74 | 0.07 | 10.62 |
| GA2 | 0.54 | 0.06 | 8.82 | 0.73 | 0.06 | 11.60 | 0.02 | 0.05 | 0.41 | 0.71 | 0.07 | 10.34 | 0.75 | 0.08 | 9.69 |
| GA3 | 0.50 | 0.07 | 7.15 | 0.68 | 0.09 | 7.84 | 0.11 | 0.11 | 0.95 | 0.78 | 0.09 | 8.81 | 0.57 | 0.11 | 5.08 |
| Core (ρ = .70) |  |  |  |  |  |  |  |  |  |  |  |  |  |  |  |
| GA4 | 0.27 | 0.10 | 2.67 | 0.42 | 0.16 | 2.65 | 0.06 | 0.12 | 0.52 | 0.49 | 0.20 | 2.45 | 0.36 | 0.17 | 2.10 |
| GA5 | 0.32 | 0.11 | 2.81 | 0.45 | 0.16 | 2.82 | 0.09 | 0.13 | 0.66 | 0.37 | 0.20 | 1.83 | 0.54 | 0.16 | 3.29 |
| GA6 | 0.50 | 0.05 | 9.11 | 0.77 | 0.07 | 11.48 | 0.05 | 0.11 | 0.49 | 0.82 | 0.09 | 8.79 | 0.71 | 0.10 | 7.11 |
| GA7 | 0.47 | 0.06 | 7.66 | 0.76 | 0.07 | 11.33 | 0.03 | 0.08 | 0.39 | 0.73 | 0.08 | 9.28 | 0.79 | 0.10 | 8.16 |
| Anxiety *(ρ = .83)* |  |  |  |  |  |  |  |  |  |  |  |  |  |  |  |
| A1 | 0.17 | 0.03 | 5.54 | 0.52 | 0.08 | 6.43 | 0.02 | 0.08 | 0.19 | 0.53 | 0.12 | 4.58 | 0.50 | 0.09 | 5.58 |
| A2 | 0.17 | 0.04 | 4.61 | 0.50 | 0.09 | 5.81 | 0.10 | 0.18 | 0.55 | 0.40 | 0.16 | 2.53 | 0.59 | 0.13 | 4.43 |
| A3 | 0.25 | 0.03 | 9.45 | 0.72 | 0.05 | 15.80 | 0.07 | 0.10 | 0.68 | 0.78 | 0.08 | 9.35 | 0.65 | 0.07 | 9.94 |
| A4 | 0.25 | 0.02 | 10.82 | 0.78 | 0.04 | 20.49 | 0.01 | 0.07 | 0.20 | 0.79 | 0.07 | 10.91 | 0.77 | 0.05 | 14.10 |
| A5 | 0.25 | 0.03 | 9.87 | 0.73 | 0.05 | 15.53 | 0.03 | 0.09 | 0.36 | 0.77 | 0.09 | 8.99 | 0.70 | 0.09 | 8.21 |
| A6 | 0.17 | 0.03 | 5.99 | 0.51 | 0.08 | 6.67 | 0.03 | 0.08 | 0.45 | 0.54 | 0.09 | 5.87 | 0.47 | 0.09 | 5.11 |
| A7 | 0.25 | 0.02 | 11.47 | 0.74 | 0.05 | 15.69 | 0.08 | 0.10 | 0.77 | 0.66 | 0.09 | 7.33 | 0.82 | 0.05 | 14.87 |
